# Antibody response and cross-neutralization after Omicron BA.2 infection

**DOI:** 10.1038/s41392-022-01305-3

**Published:** 2023-01-07

**Authors:** Yiwen Zhang, Rong Li, Yuzhuang Li, Hong Yang, Liqiong Zhou, Jing Yuan, Ting Pan, Bingfeng Liu, Hui Zhang, Yaqing He

**Affiliations:** 1 Institute of Human Virology, Key Laboratory of Tropical Disease Control of Ministry of Education, Guangdong Engineering Research Center for Antimicrobial Agent and Immunotechnology, Zhongshan School of Medicine, Sun Yat-sen University, Guangzhou, Guangdong 510080 China; 2 Shenzhen Center for Disease Control and Prevention, Shenzhen, 518055 Guangdong China; 3 Shenzhen Third People’s Hospital, Second Hospital Affiliated to Southern University of Science and Technology, South China Hospital of Shenzhen University, Shenzhen, 518011 Guangdong China

**Keywords:** Microbiology, Infectious diseases

**Dear Editor**,

The Omicron (B.1.1.529) variant of severe acute respiratory syndrome coronavirus 2 (SARS-CoV-2) was first identified in November 2021, in South Africa and Botswana. The first Omicron sub-lineage that emerged was BA.1, which was supplanted by BA.2 in many countries. One of the most notable features of the Omicron variant is its ability to evade neutralizing antibodies (nAbs) targeting the original virus lineages, owing to new mutations dotted among the spike protein, especially in the receptor-binding domain (RBD) and N-terminal domain.^1,2^ Therefore, the immune response arising from Omicron in individuals who have already been inoculated with vaccines is a cause of concern. However, to date, little is known about how the Omicron/BA.2 variant interacts with vaccine-induced immunity to affect the infection. In this study, we collected sera from individuals in the acute or convalescent phase after BA.2-breakthrough infection and then detected levels of antibodies and their neutralizing ability, followed by cross-assessments among different variants.

First, we enrolled 31 volunteers confirmed as Omicron BA.2 cases via genome sequencing performed by the Centers for Disease Control (CDC, Shenzhen). Among them, 14 were fully vaccinated with the CoronaVac vaccine before BA.2 infection, 13 had received the third dose (booster) before BA.2 infection, and four were unvaccinated and infected with BA.2. Serum samples were collected within 3 days after diagnosis. To investigate the early humoral immune response, titers of specific antibodies were first measured, and cross-reactivity was detected. In agreement with previous reports regarding the enhanced immune-escape ability of Omicron, the early antibody response was skewed toward ancestral SARS-CoV-2 strains.^1^ Further, the booster (third) dose of the vaccine resulted in a markedly higher titer of IgG, which not only significantly bound the BA.2 spike protein but also had high affinity for the D614G and Delta spike proteins (Fig. 1a). Furthermore, after processing pseudotyped virus assay results, we found that individuals vaccinated with either two or three doses had apparently more potent nAbs^3^ (Fig. 1b). Meanwhile only one of four unvaccinated volunteers had nAbs at a detectable level, whereas samples from 8 of 14 and 10 of 13 volunteers in the fully vaccinated and boosted groups, respectively, showed marked neutralizing capacity (Fig. 1b). These results suggested that the third (booster) vaccine dose provided better protection against BA.2 at the early phase.

**Fig. 1:**
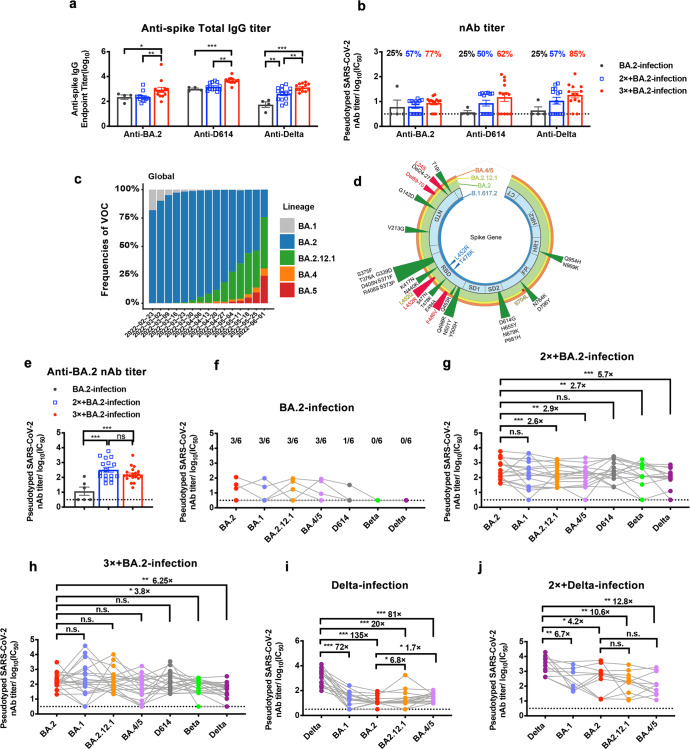
Antibody response and cross-neutralization after Omicron BA.2 Infection. **a**, **b** Serum samples of BA.2-infected individuals were collected within 3 days of the first positive PCR test confirmation. Individuals infected with BA.2 (*n* = 4), individuals who received two doses of the CoronaVac vaccine prior to BA.2 infection (*n* = 14), and individuals who received three doses of the CoronaVac vaccine prior to BA.2 infection (*n* = 13) were included. **a** Specificity to spike variants (BA.2, D614, and Delta) and levels of antibodies in the acute phase in each of the three cohorts were measured via ELISA and are shown as the endpoint titer. **b** Neutralizing abilities were detected by incubating serially diluted serum samples with pseudotyped SARS-CoV-2 variants (BA.2, D614, and Delta), and the NT50 was calculated by measuring the luciferase activity of infected hACE2-293T cells. **c** Frequencies of BA.1, BA.2, BA.2.12.1, BA.4, and BA.5 deposited in GISAID. **d** Schematic of the Spike gene of SARS-CoV-2 variants, which included Delta (B.1.617.2, blue circle), BA.2 (green circle), BA.2.12.1 (yellow circle), and BA.4/5 (orange circle). Common mutations among three Omicron subvariants are marked as green triangles and indicated with black labels. Characteristic mutations in BA.2.12.1 or BA.4/5 are marked as red triangles and indicated with yellow (BA.2.12.1) or red (BA.4/5) labels. Mutations in Delta (B.1.617.2) are marked as blue triangles and labels. **e** Neutralizing titers against Omicron BA.2 pseudoviruses in sera from BA.2 convalescent individuals. **f–h** Neutralizing titers against Omicron sub-lineages and SARS-CoV-2 D614, Beta, and Delta pseudoviruses in sera from BA.2 convalescent individuals. **f** Individuals infected with BA.2 (*n* = 6). **g** Individuals who received two doses of the CoronaVac vaccine prior to BA.2 infection (*n* = 17). **h** Individuals who received three doses of the CoronaVac vaccine prior to BA.2 infection (*n* = 20). **i**, **j** Neutralizing titers against Omicron sub-lineages and SARS-CoV-2 D614, Beta, and Delta pseudoviruses in sera from Delta convalescent individuals. **i** Individuals infected with Delta (*n* = 15). **j** Individuals who received two doses of the CoronaVac vaccine prior to Delta infection (*n* = 11). Dashed lines indicated the limit of detection. Data were analyzed by performing a Kruskal–Wallis comparisons test (**a**, **b**). *P* values were calculated using two-tailed Wilcoxon signed-rank tests of paired samples (**g**–**j**). *p < 0.05, **p < 0.01, ***p < 0.001, n.s. not significant. All neutralization assays were conducted with biological duplicates

Globally, reported BA.2 cases have rapidly surpassed those of BA.1. Recently, the detection and subsequently pronounced prevalence of three newly emerging sub-lineages, BA.2.12.1, BA.4, and BA.5, have raised concerns (Fig. 1c). BA.2.12.1, which contains an identical RBD protein sequence with that of BA.2, other than an additional L452Q mutation, was first reported in the United States in February 2022 (Fig. 1d). The spike proteins of BA.4 and BA.5 were found to be identical, with four additional mutations in BA.2 as follows: Del69-70, L452R, F486V, and R493Q (Fig. 1d). BA.4 and BA.5 variants were primarily reported in South Africa in January, and related cases rapidly increased, with a combined frequency of greater than 30% for all new Omicron infections globally (Fig. 1c). These mutations mostly endow the emerging variants with nAb-evasion capacity. We further enrolled 43 volunteers and divided them into three groups as follows: (a) individuals infected with BA.2 and those who received (b) two or (c) three doses of inactive-virus vaccine prior to BA.2-breakthrough infection. All cases were confirmed via sequencing performed by the CDC, Shenzhen, and sera were obtained after day 28 of the first PCR confirmation. Similarly, we evaluated neutralizing potency using pseudotyped virus assays. First, we compared the BA.2 neutralization ability using serum samples from the two groups, vaccinated or unvaccinated individuals. As hypothesized, samples obtained from the unvaccinated group had neutralization activity with low nAb titers^4^ (Fig. 1e–h and supplementary Fig. S1). In the vaccinated groups, BA.2nAbs were increased by more than 15-fold, and this was independent of the boosting status (Fig. 1e). Regarding the neutralizing potency against all Omicron sub-lineages, the lowest activity was against BA.4/5, followed by higher activity against BA.2.12.1, which were decreased by 2.9-fold and 2.6-fold, respectively, relative to that found in the BA.2-breakthrough cohort with two vaccine doses (Fig. 1g). Notably, sera from individuals who had received three doses of CoronaVac before BA.2-breakthrough infection did not display significantly impaired potency in the BA.4/5 and BA.2.12.1 pseudotyped virus assays (Fig. 1h). Moreover, we probed the neutralizing ability of the sera against previous variants of concern (VOCs), including the Beta and Delta strains. As a result, unlike that in the unvaccinated group, in which the antibody response was skewed heavily toward the Omicron strain/sub-lineages, the vaccinated groups showed the considerable neutralizing ability that protected the host from D614, Beta, and Delta strain infections, suggesting that the effectiveness of and significance of memory humoral immunity conferred by the preceding vaccination (Fig. 1f–h). Remarkably, the nAb titer against Delta was significantly lower when compared to that against Beta, showing a reduction of 5.7-fold and 6.25-fold, as compared to the BA.2 nAb titer, whereas the titer of nAbs against Beta was reduced by 2.7-fold and 3.8-fold, when compared with the BA.2 nAb titer, in the two- and three-dose groups, respectively (Fig. 1g, h). Altogether, these data indicate that the newly emerging BA.2.12.1 and BA.4/5 strains might give rise to re-infection because of their marked ability to evade previously acquired immunity.

The Delta strain and BA.4/5 and BA.2.12.1 sub-lineages harbor an identical mutation at residue 452, a key residue that was reported to facilitate immune evasion against previously reported nAbs (Fig. 1d). Subsequently, we evaluated the nAb resistance in 26 patients with COVID-19 during the Delta pandemic, May–June 2021, in Guangzhou.^5^ Among them, 11 were fully vaccinated with the CoronaVac vaccine and 15 were unvaccinated before Delta infection. After screening samples from these cohorts, we found that the nAbs that were generated only by the previous Delta infection showed drastically reduced activity against Omicron, suggesting a low, if any, humoral immune response to the current Omicron sub-lineages (Fig. 1i). Notably, BA.4/5 and BA.2.12.1 exhibited less resistance to neutralization by Delta convalescent serum, as evidenced by an increase in the nAb titer against BA.4/5 and BA.2.12.1, which was enhanced by 1.7-fold and 6.8-fold, respectively, compared with the BA.2 nAb titer (Fig. 1j). In line with the results using BA.2 convalescent sera and those of previous studies in which humoral immunity was improved after vaccination, Delta convalescent sera from vaccinated groups had a more than twofold higher nAb titer against the Delta pseudotyped virus (Fig. 1f–j). The nAb titer of the Delta convalescent group previously administered the vaccine was reduced by 6.7-fold, 4.2-fold, 10.6-fold, and 12.8-fold in response to Omicron sub-lineages BA.1, BA.2, BA.2.12.1, and BA.4/5, respectively, indicating certain degrees of humoral immune protection (Fig. 1j).

In this study, we characterized BA.2 infection-induced and vaccine-induced immunity against Omicron sub-lineages BA.2.12.1, BA.4, and BA.5. We found that an additional (third) vaccine booster dose results in higher anti-spike IgG and nAb titers at the early phase of the BA.2-breakthrough infection. Furthermore, we systematically assessed the immune evasion capacity of newly emerging sub-lineages of SARS-CoV-2 Omicron based on the immunity conferred by inactivated virus vaccines against BA.2 infection. Our data suggest that in the event of a BA.2-breakthrough infection, previous vaccination allows for a stronger humoral immune response with a broader spectrum neutralization response to the VOCs and the Omicron sub-lineages. In addition, mutations at residue 452 comprise one of the key drivers of the neutralization-resistance phenotype, as BA.2.12.1 and BA.4/5 were more resistant than the other strains. Because L452R is also a defining mutation of the Delta variant, we observed that the new Omicron sub-lineages, which contained mutations at 452, were more effectively neutralized by Delta convalescent sera. These results provide insights into the role of pre-existing humoral immunity when exposed to heterologous SARS-CoV-2 variants. The continuous evolution of Omicron poses a great challenge to ancestral SARS-CoV-2 vaccine-induced or BA.1/BA.2 infection-induced herd immunity.

## Supplementary information

supplementary Materials

## Data Availability

The data were available from the corresponding author upon reasonable request.
