# Supplementary material for: Antibody response and cross-neutralization after Omicron BA.2 infection
Source: Signal Transduct Target Ther. 2023 Jan 7;8:25. doi: 10.1038/s41392-022-01305-3 (PMC9823246; doi:10.1038/s41392-022-01305-3)
Supplement: Supplementary file 1 — supplementary Materials [file 41392_2022_1305_MOESM1_ESM.docx]

**Supplementary Materials for**

**Antibody Response and Cross-Neutralization After Omicron BA.2 Infection**

Yiwen Zhang^1,*^, Rong Li^1,*^, Yuzhuang Li^1,*^, Hong Yang^2^, Liqiong Zhou^3^, Jing Yuan^4^, Ting Pan^1,5^, Bingfeng Liu^1, #^,Hui Zhang^1,#^, and Yaqing He^2,#^.

**This PDF file includes:**

Supplemental figure

Methods

**Supplemental Figure**

**Fig S1. Neutralizing ability After Omicron BA.2 Infection**

Neutralizing titers against Omicron sub-lineages and SARS-CoV-2 D614, Beta, and Delta pseudoviruses in sera from BA.2 convalescent individuals. Individuals who received two-dose of CoronaVac vaccine prior to BA.2 infection (n=17). Individuals who received three-dose of CoronaVac vaccine prior to BA.2 infection (n=20). Dash lines indicated the limit of detection. Data were analyzed by Mann-Whitney t test. n.s., not significant. All neutralization assays were conducted in biological duplicates.

**Methods**

**Plasmids construction.**

SARS-CoV-2 spike variants-expressing plasmids were constructed to package pseudotyped SARS-CoV-2 S/HIV-1 viruses as previously described^1,2^. The gene encoding the spike protein of the D614 virus (Wuhan-Hu-1, GISAID: EPI_ISL_402125) was codon-optimized and cloned into pcDNA3.1 vector, as well as the other variants including BA.2 (GISAID: EPI_ISL_13199355), BA.1 (GISAID: EPI_ISL_13199205), BA.2.12.1 (GISAID: EPI_ISL_13199386), BA4/5 (GISAID: EPI_ISL_13199107), Beta (GISAID: EPI_ISL_13183683) and Delta (EPI_ISL_13159105 ). The sequences of all the spike protein-expressing plasmids were verified by Sanger sequencing.

**Pseudotyped virus neutralization assay.**

Pseudotyped SARS-CoV-2 S/HIV-1 viruses were packaged in HEK293T cells by co-transfecting with a lentiviral construct pHIV-Luciferase (Addgene plasmid # 21375), a packaging construct psPAX2 (Addgene plasmid # 12260) and a pcDNA3.1 vector-plasmid expressing spike variants including D614 virus, BA.2, BA.1, BA.2.12.1, BA.4/5, Delta, Beta^2,3^. The culture medium was replaced with fresh DMEM supplemented with 10% FBS and 1% penicillin-streptomycin 6 hours post transfection. The supernatantwas collected 48 hours post transfection. The pseudotyped virus-containing supernatant was stored at -80℃ and titrated before neutralization assays.

For detecting neutralizing potency against pseudotyped SARS-CoV-2 variants, serially diluted plasma from heathy individuals, COVID-19 acute-phase patients and convalescent individuals were mixed with virus and incubated at 37℃, 5% CO2 for 1 h. Plasma/virus mixtures were added into 96-well plates seeded with 1×10^4^ hACE2-HEK293T cells/well and cultured for 48 h. After discarding the supernatant, cells were lysed with passive lysis buffer (Promega) and the lysate was measured for relative luminescence units with luminometer (Promega). Neutralizing antibodies titers of plasma against indicated pseudotyped viruses were analyzed with GraphPad Prism 8.0 software using non-linear regression to measure the NT50 titer. The percent inhibition of diluted sera was calculated by analyzing the RLU value via the following formula: 100-((([sample well]-[average of "cell control" wells])/([average of "virus control” wells]-[average of "cell control" wells))*100). The NT50 titer was defined as the reciprocal of serum dilution at which nAbs caused 50% inhibition of infection. The cut-off value is the 50% inhibition value. Before performing neutralization assays against different variants, we titrated each variant of pseudotyped virus to calculate their TCID. When detecting neutralization activity of sera, these titrated pseudotyped virus were diluted to achieve a TCID of approximately 1000000-5000000 Relative Luminescence Units (RLU) for normalizing the infection efficacy.

**Plasma IgG detection.**

SARS-CoV-2 S1+S2 ECD proteins (Sino Biological Inc., 40589-V08H28, 40589-V08B1 and 40589-V08B16) (1.5 μg/ml) were coated onCostar Stripwell^TM^ Microplates at 4 °C overnight (50 μl/well). The plates were blocked with 5% non-fat milk in PBS for 2 hours at 37 °C. Subsequently the plates were washed with PBS c followed by incubating with 6-fold serially diluted plasma in PBS for 1 hour at 37 °C. After washing three times with 0.05% PBS-T, HRP-conjugated goat anti--human IgG secondary antibody (abcam, ab7153) was added and incubated for 1 hour at 37 °C. The plates were then washed four times with PBS-T, followed by adding 50 μl of tetramethylbenzidine (TMB) substrate (Invitrogen) at room temperature protected from light. After 10 minutes of incubation, the reaction was stopped with 50 μl 2 M H_2_SO_4_ solution. The absorbance of each well was measured at 450 nm.

**Statistical analysis.**

All the measurements have been performed for at least three times by at least two laboratory technicians. Detailed statistical information for specific experiments, including statistical tests, number of samples, mean values, standard errors of the mean (SEM) and p-values derived from indicated test, had been annotated in the main text, figure legends and showed in the figures. Statistical analysis was conducted with Graphpad Prism 8.0 or Microsoft Excel. A value of p ≥ 0.05 was considered to be not statistically significant and represented as “n.s.”. A value of p < 0.05 was considered to be statistically significant and represented as asterisk (*). Value of p < 0.01 was considered to be more statistically significant and represented as double asterisks (**). Value of p < 0.001 was considered to be the most statistically significant and represented as triple asterisks (***).

**Reference**

1. Ma, X., Zou, F., Yu, F., Li, R., Yuan, Y. *et al.* Nanoparticle Vaccines Based on the Receptor Binding Domain (RBD) and Heptad Repeat (HR) of SARS-CoV-2 Elicit Robust Protective Immune Responses. *Immunity.* 53, 1315-1330.e9 (2020).

2. Yuan, Y., Zhang, X., Chen, R., Li, Y., Wu, B. *et al.* A bivalent nanoparticle vaccine exhibits potent cross-protection against the variants of SARS-CoV-2. *Cell Rep.* 38, 110256 (2022).

3. Zhang, Y., Chen, Y., Li, Y., Huang, F., Luo, B. et al. The ORF8 protein of SARS-CoV-2 mediates immune evasion through down-regulating MHC-Ι. Proc Natl Acad Sci U S A. 118, e2024202118 (2021).
